# You are welcome here: A practical guide to diversity, equity, and inclusion for undergraduates embarking on an ecological research experience

**DOI:** 10.1002/ece3.7321

**Published:** 2021-03-10

**Authors:** Bonnie M. McGill, Madison J. Foster, Abagael N. Pruitt, Samantha Gabrielle Thomas, Emily R. Arsenault, Janaye Hanschu, Kynser Wahwahsuck, Evan Cortez, Kaci Zarek, Terrance D. Loecke, Amy J. Burgin

**Affiliations:** ^1^ Kansas Biological Survey University of Kansas Lawrence KS USA; ^2^ Land Resources and Environmental Sciences Montana State University Bozeman MT USA; ^3^ Department of Biological Sciences University of Notre Dame Notre Dame IN USA; ^4^ Environmental Studies Program University of Kansas Lawrence KS USA; ^5^ Ecology and Evolutionary Biology Department University of Kansas Lawrence KS USA

**Keywords:** diversity, ecology, equity, inclusion, undergraduate research experience

## Abstract

As we build a more diverse, equitable, and inclusive culture in the ecological research community, we must work to support new ecologists by empowering them with the knowledge, tools, validation, and sense of belonging in ecology to succeed. Undergraduate research experiences (UREs) are critical for a student's professional and interpersonal skill development and key for recruiting and retaining students from diverse groups to ecology. However, few resources exist that speak directly to an undergraduate researcher on the diversity, equity, and inclusion (DEI) dimensions of embarking on a first research experience. Here, we write primarily for undergraduate readers, though a broader audience of readers, especially URE mentors, will also find this useful. We explain many of the ways a URE benefits undergraduate researchers and describe how URE students from different positionalities can contribute to an inclusive research culture. We address three common sources of anxiety for URE students through a DEI lens: imposter syndrome, communicating with mentors, and safety in fieldwork. We discuss the benefits as well as the unique vulnerabilities and risks associated with fieldwork, including the potential for harassment and assault. Imposter syndrome and toxic field experiences are known to drive students, including students from underrepresented minority groups, out of STEM. Our goal is to encourage all students, including those from underrepresented groups, to apply for UREs, build awareness of their contributions to inclusion in ecology research, and provide strategies for overcoming known barriers.

## Graphical abstract

Undergraduate research experiences (URE) benefit students in a variety of ways, including students from underrepresented groups in STEM. URE students from different positionalities can contribute to an inclusive research culture. We address three common sources of anxiety for URE students through a DEI lens: imposter syndrome, communicating with mentors, and safety in fieldwork.

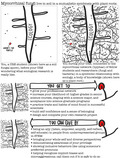

## 1 INTRODUCTION

Undergraduate research experiences (UREs) are essential for young ecologists to gain skills applicable to many postbaccalaureate jobs, be competitive for graduate level ecology programs, and develop a sense of self‐efficacy and belonging in ecology (Graham et al., 2013; Hernandez et al., 2018; Linn et al., 2015; National Academies of Sciences, 2017). UREs are critical opportunities for first‐generation students (students who are the first in their immediate family to attend college) to gain insight into the research process and culture. Ecologists are working to identify barriers to more inclusive and equitable research practices and develop a culture that welcomes and retains a more diverse community, for example, Clancy et al. (2020), Schneider et al. (2018), and Tseng et al. (2020). One element of that work is the development of inclusive and equitable UREs for students from underrepresented groups—communities historically excluded due to structural barriers in US higher education.

Here, we use the phrase “underrepresented groups in science, technology, engineering, and math (STEM)” to refer to minoritized groups, including Black, Indigenous, and People of Color or BIPOC, ethnicities, genders, sexualities, and economic classes, as well as individuals who are first generation and/or an intersection of these identities. People in these groups are underrepresented among STEM students and faculty compared to the US population (Bernard & Cooperdock, 2018; Marginson, 2016; Ong et al., 2011; US Department of Education, 2018; US National Science & Foundation, 2019). Greater diversity is essential for building a community of ecologists that reflects the global communities we aim to serve and spurs greater innovation (Ferrini‐Mundy, 2013), both of which are critical for addressing the complex socioecological issues of the 21st century.

Ideally, URE students develop the ability to interpret their data and generate new research questions; however, a barrier to attainment is the length of time it takes for students to catch up to the culture of scientific practices (Linn et al., 2015). Many publications describe strategies for how mentors can make their laboratory culture and UREs more equitable and inclusive, for example, Nocco et al. (2021), Dodson et al. (2009), Montgomery (2017), Montgomery (2018), Puniwai‐Ganoot et al. (2018), Demery and Pipkin (2020), Emery et al. (2019), and Emery et al. (2020). However, few resources exist that speak directly to an undergraduate researcher on the diversity, equity, and inclusion (DEI) dimensions of embarking on a first research experience. We, the co‐authors, are a group of four recent or current undergraduates and seven graduate students and scientists, including an individual who self‐identifies as Kiikaapoa/Shoshone/Sauk and Fox and one or more individuals who self‐identify as: a veteran, LGBTQ+, first generation, and from a working class background. We acknowledge that we, except our Kiikaapoa/Shoshone/Sauk and Fox co‐author, benefit from the power and privilege that comes from being white. We cannot speak for all the dimensions of diversity, but in writing this paper, all co‐authors “strive to be accomplices, co‐conspirators, and allies to and with marginalized and underserved groups in science through meaningful action to promote inclusivity” (Emery et al., 2020). More specifically, we aim for undergraduate readers of all identities to feel welcome in ecology and be well‐informed and better prepared for a successful URE. The following sections describe (a) why an undergraduate student, “you,” should apply for a URE, (b) how URE students with different positionalities can contribute to an inclusive lab culture, and (c) information for navigating potential URE hurdles like imposter syndrome, communicating with your advisor, and safety in fieldwork.

## 2 WHY YOU SHOULD APPLY FOR A URE

**FIGURE 1:**
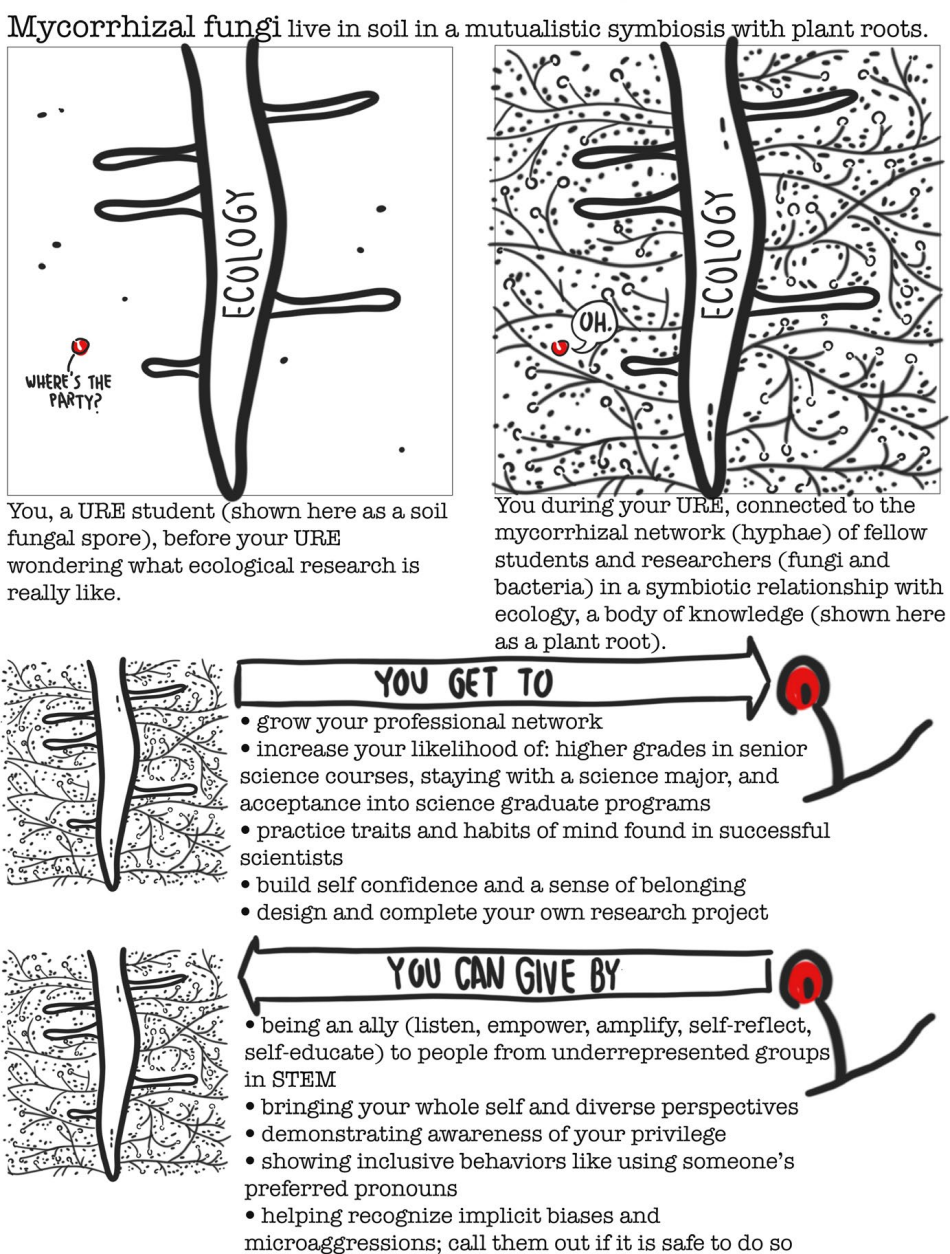
The undergraduate research experience (URE) is analogous to mycorrhizal fungi in symbiosis with a plant. In a URE, the contributions of the research mentor and community to the student's development are expected to outweigh the optional contributions listed here that the student can make to the laboratory culture. Original artwork

A URE is a bit of a symbiosis, like mycorrhizal fungi. Mycorrhizal fungi live in soil in a mutualistic symbiosis with plant roots, which provide the fungi with carbon, and the fungi provide plant roots with soil minerals. In this section, we describe the benefits you get from a URE in ecology, similar to the carbon a mycorrhizal fungi receive from a plant root (Figure 1). UREs improve students’ likelihood of success later in their undergraduate careers and beyond. Our understanding of how a URE can impact career outcomes materializes from more generalized STEM disciplines (e.g., biology or geosciences, instead of a sub‐discipline like ecology). Undergraduate STEM majors who complete a high contact (10+ hours per week) URE are, compared to students who do not do a URE, more likely to earn higher grades in senior science courses, be accepted into science graduate programs, and be engaged in a scientific career or scientific training six years after graduation (Hernandez et al., 2018; Linn et al., 2015). A URE can reconnect you with your initial inspiration to be a scientist, despite potentially uninspiring introductory courses (Graham et al., 2013). Your URE will help you identify gaps in your knowledge and, thus, the future courses you might like to take (Linn et al., 2015), potentially helping you find continuity between your interest, experience, courses, and career goals. Participating in a URE in the first two years of college increases the likelihood you will persevere with your science major, particularly if you are from an underrepresented group (Graham et al., 2013).

In order to achieve the above measures of success, you will build a surprising number of personal and interpersonal skills. Every scientist is different, but most successful scientists embody traits such as curiosity, patience, perseverance, and initiative (Graham et al., 2013; Hernandez et al., 2018; Linn et al., 2015). Social and cultural processes that science relies on include collaboration, critique, collegiality, mentorship, and peer review (National Academies of Sciences, 2017). All of these traits can be developed with practice, and a URE provides ample training to flex these skills, most of which are difficult to learn through coursework or reading alone. During your URE you will start to build the above traits; participate in the above processes; and learn the habits of mind, values, norms, and practices of researchers (National Academies of Sciences, 2017). A URE gives you a backstage pass to see how scientific research actually works and feels. In the process, you will start to integrate background information and ecological concepts, update them with your observations and results, and develop an evidence‐based argument (Linn et al., 2015). By working with complicated research topics, persisting through countless rounds of trial and error, and owning your contributions to the laboratory group, you will build confidence in yourself as a scientist (Graham et al., 2013; Linn et al., 2015). Through a URE, you will expand your professional network (Figure 1), which can lead to future employment.

On a more personal level, a URE is an opportunity to “try on” ecology to see whether it aligns with your expectations, values, and goals (Linn et al., 2015). Through the experience, you will build a professional identity as a scientist and an understanding of how your own personality aligns with the traits needed to conduct science. Ideally, a URE will also provide you with a sense of belonging and inclusion in STEM, which is especially critical for students from underrepresented groups (National Academies of Sciences, 2017). URE mentors and their laboratory groups have the ability to cultivate an inclusive and equitable URE for all students (see Box 1).

BOX 1 **Suggestions for mentors to make UREs in ecology more equitable and inclusive**

System change starts with those in leadership positions. Here is a brief list of ways principal investigators (PIs) and other URE mentors, "you" in this box, can cultivate an inclusive and equitable laboratory team ethos. Such a laboratory builds allyship in students and staff of privilege and values and accommodates the varieties of experiences and vulnerabilities of students from underrepresented minorities.

- Transparency, open‐mindedness, compassion, and a growth mindset nurture a trusting relationship based on respect where the student can bring their full self—not assimilate or hide their identities (Nocco et al., 2021; Emery et al., 2020; Montgomery, 2018). Create an atmosphere for your laboratory community to discuss DEI topics, for example, start by reading and discussing a DEI‐related paper as a laboratory group.
- Considering how exclusion and imposter syndrome impact retention of underrepresented students in STEM (Arnold et al., 2020), structure your laboratory's undergraduate research experience to validate students’ abilities, build confidence, and a sense of belonging in ecology. Praise from a PI may be especially powerful because the student perceives the PI to have greater knowledge and experience (Joshi et al., 2019). Share a recent failure of yours with the laboratory group.
- PIs should familiarize themselves with best hiring practices, including advertisement language, avoiding implicit biases, and being flexible with prerequisites—look for evidence the student has key traits like motivation and curiosity. Avoid using arbitrary test scores or grade cutoffs for selection (Emery et al., 2020). A lack of prior experience with or appropriate gear for field activities should not exclude students from URE selection.
- Develop custom laboratory and field codes of conduct, for example, see ADVANCEGeo Partnership (2020). PIs and laboratory team members can share the burden of differential risk to underrepresented minority students from the public during fieldwork by educating themselves on the risks and strategies for mitigating risk (we highly recommend Demery & Pipkin, 2020). Equity in safety does not mean treating all laboratory members the same when risks are different; it means one‐on‐one discussions of risk with at‐risk individuals are an inclusive practice (Demery & Pipkin, 2020). Emphasize that researcher safety is more important than the data.
- Learn to say students’ names properly and use their preferred pronouns (Emery et al., 2020).
- If a student shares with you that they have been harassed or assaulted, please remember three things: (a) You likely are a mandatory reporter and need to follow institutional guidance; (b) it is not your role to decide whether their experience was or was not harassment or assault; (c) once the case is reported to the Title IX office, their trauma is not necessarily over, and you should continue to offer support. Share with the student information about counseling services (including nonmandatory reporters) available to them at your institution.
- Consider attending or organizing an implicit bias training to help your community become more effective at doing ally work (e.g., ADVANCEGeo Partnership workshops https://serc.carleton.edu/advancegeo/workshops/).

When looking for UREs, shop around. Just as consumer demand can change business practices, so too can informed UREs nudge research laboratories to provide equitable and inclusive experiences. Look for UREs that are paid. Volunteer‐based UREs are not equitable because they exclude people who cannot afford to work for free. Look for laboratories that have codes of conduct; have an individual accountable for your mentorship (such as the principal investigator or a graduate student) who has undergone DEI mentorship training; have diverse laboratory members; give credit to undergraduate researchers on their laboratory website; state their commitment to DEI on their laboratory website; include URE students in laboratory meetings; and potentially send URE students to symposia to present their research. If your URE is at a different institution (e.g., a summer internship), look for ones that (or ask that they) cover your travel costs up front as opposed to reimbursing you later and provide student housing and professional development opportunities. URE positions can be competitive, so you might not be able to be very picky, but these are helpful things to know to look for.

## 3 HOW YOU CAN CONTRIBUTE TO DIVERSITY, EQUITY, AND INCLUSION IN ECOLOGY RESEARCH CULTURE

To students who identify with any minority groups or intersection of identities underrepresented in ecology: *You belong here!* Please know that there is a loud group of scientists advocating for you to join ecology and reach positions of influence, offering advice on how to succeed, and how scientists need to change the research system to make it more equitable and inclusive, for example, Beltran et al. (2020), Clancy et al. (2020), Demery and Pipkin (2020), Halsey et al. (2020), Marín‐Spiotta et al. (2020), and Tseng et al. (2020). To students who identify with overrepresented identities in ecology (e.g., white, men, able‐bodied, and/or heterosexual individuals): *You belong here!* You have an important role to play in making the culture of STEM more inclusive. We acknowledge that the majority of the responsibility to cultivate an inclusive and equitable research community ethos is on laboratory leadership and their institutions. The aim of this section is to assist all undergraduate researchers in building awareness of some of the DEI challenges you may see or experience, as well as the ways you can contribute to an inclusive research culture. Going back to the fungi–plant symbiosis, what you can give to the ecology research culture is like the fungi bringing new minerals to the plant root (Figure 1).

Each of us has a unique combination of identities that put us somewhere on a spectrum between privileged and underprivileged. A first step toward personally contributing to an inclusive laboratory culture is to recognize your own privilege and positionality (i.e., your privilege relative to someone else). We offer some places to start in Table 1A. Confronting your privilege can be uncomfortable, but it is important to work through these feelings for your own wellbeing as well as those around you. Remember to separate your privilege from your identity as a good person—the two are not mutually exclusive (Dutt, 2020). Awareness of our privilege and positionality has implications for how we interact with others, for example, this may be your first time working with someone you have more or less privilege than—do you treat them differently?

**TABLE 1:** Resources for DEI support and awareness

A. Resources for DEI support and awareness	
Building awareness of diverse identities: https://pflag.org/, https://www.racialequitytools.org/home, https://diversity.berkeley.edu/ei‐archive, https://www.complex.com/pop‐culture/the‐best‐black‐movies‐of‐the‐last‐30‐years/, https://www.stemwomen.net/, https://diversity.nih.gov/, https://lgbtq.arizona.edu/resources, https://www.nature.com/articles/d41586‐021‐00022‐1 • Privilege: http://also‐chicago.org/also_site/wp‐content/uploads/2017/03/white‐privilege.pdf, https://edge.psu.edu/workshops/mc/power/index.html • Implicit bias: http://www.eigenfactor.org/gender/, https://blogs.lse.ac.uk/impactofsocialsciences/2016/03/08/gender‐bias‐in‐academe‐an‐annotated‐bibliography/ • Microaggressions: https://youtu.be/crAv5ttax2I, https://nyti.ms/2wsh0bY, https://youtu.be/BJL2P0JsAS4, https://youtu.be/hDd3bzA7450, https://youtu.be/KPRA4g‐3yEk, https://www.lifescied.org/doi/10.1187/cbe.18‐01‐0011 • Support for underrepresented scientists (also see groups above): https://www.nature.com/articles/s41559‐020‐1252‐0, https://www.chronicle.com/article/A‐Survival‐Guide‐for‐Black/249118 • Allyship for nonunderrepresented scientists: https://doi.apa.org/fulltext/2019‐01033‐011.html, https://www.lifescied.org/doi/10.1187/cbe.20‐04‐0062, https://www.wbur.org/artery/2020/06/17/reading‐list‐on‐race‐for‐allies	
B. Some of the many groups supporting different facets of DEI in STEM	
BLACK and STEM	A community advocating for black students and professionals in STEM fields (Twitter: @BLACKandSTEM)
FirstGenDocs	A group that celebrates the experiences and voices of first‐generation doctoral students and graduates (Twitter: @firstgendocs)
500 Queer Scientists	A visibility campaign for the LGBTQ+ STEM community (Twitter: @500QueerSci)
500 Women Scientists	A diverse group of women scientists with a commitment to foster real change in DEI (Twitter: @500womensci)
Black AF in STEM	A community aiming to showcase the experiences of Black scientists (Twitter: @BlackAFinSTEM)
Black Women in STEM	A group that connects black women in STEM fields (Twitter: @BlackWomenSTEM)
Científico Latino	A resource for undergraduate and graduate students in STEM (Twitter: @cientificolatin)
ESA SEEDS	Strategies for Ecology Education, Diversity and Sustainability (SEEDS) is a minority education and mentoring program of the Ecological Society of America (Twitter: @ESA_SEEDS)
I’m First!	An account that celebrates and supports students who are the first in their family to attend and graduate from college (Twitter: @ImFirstGen)
LGBTQ+ STEM	A group working to improve LGBTQ+ visibility in STEM (Twitter: @LGBTSTEM)
Me Too STEM	A group invested in holding science societies accountable for harassment and discrimination (Twitter: @MeTooSTEM)
SACNAS	SACNAS is professional Society Advancing Native Americans and Chicanos/Hispanics in Science. SACNAS also hosts the National Diversity in STEM Conference each year (Twitter: @sacnas)
The EEB_POC Project	A project compiling research papers published by Black, Indigenous, and people of color scientists in Ecology, Evolution, & Behavior (Twitter: @EEB_POC)
C. Twitter hashtags to search	
#AcademicChatter #BlackintheIvory #BlackandSTEM #firstgendoc #firstgenSTEM #LGBTinSTEM #NativeTwitter #Indigenous #TEK #NativesinSTEM #phdlife #QueerInSTEM #WomenInSTEM	

A second step toward inclusion is recognizing when laboratory culture can reinforce the feeling of not belonging in subtle ways. Here are a few examples of subtle ways you, as an undergraduate student, may have the opportunity to create an inclusive environment. Mix up the music played and topics of casual conversation in research spaces (laboratories, vehicles, and field sites) to give all laboratory members the opportunity to feel culturally seen and heard to the degree they prefer. You can normalize acceptance of all levels of experience. For example, if a fellow URE student has never tried something, avoid exclamations of shock (e.g., “You've *never* skied?!”), and instead ask them what they like to do in the winter time. Another example of an everyday inclusive practice is learning to say people's names correctly and using preferred pronouns (Emery et al., 2020).

Educating yourself on implicit biases is a third step toward personally contributing to an inclusive lab. Implicit biases are “prejudices, beliefs, or attitudes toward a person or group that are not within the margins of awareness, and are thus, unconscious” making them hard to acknowledge and control (Yarber, 2020). Implicit biases are the result of living in a system of advantage and social conditioning (Dutt, 2020). We strongly suggest you explore your own implicit biases at Project Implicit https://implicit.harvard.edu/implicit/takeatest.html and see Table 1A for ways to address them.

Implicit biases are harmful, in part, because they can manifest as microaggressions. Microagressions are subtle, unconscious, automatic words, or actions that may unintentionally invalidate or insult someone because of their minority identity(ies) (Sue et al., 2007). For example, be aware of what your wording may imply: If you ask someone who is a person of color or who has an accent, “Where are you from?”, your question implies they are an outsider (an implicit bias), which can reinforce their feelings of not belonging in the space (a microaggression). Instead, get to know them by saying, “So, tell me something about yourself.” Your intention in the former question may be well meaning, but impact overrules intention (Utt, 2013). If you are the target of a microaggression, learning about the different types of microaggressions can provide you with additional language to discuss your experience and affirm that your experience is real (see Nadal (2014)). Experts suggest you protect your emotional wellbeing by choosing your battles, assessing the risk of physical danger if you respond, focusing responses on the behavior and not the person, asking clarifying questions, and seeking support (Nadal, 2014; Tseng et al., 2020).

Fourth, if you are not from an underrepresented group, learn about the different types of microaggressions and consider striving for allyship (Figure 1 and Table 1A). An ally listens more than they speak, amplifies the voices of the unheard, empowers without rescuing, and is open to being confronted about their own behavior and attitudes (Center for Community Health & Development, 2019; Lamont, 2019). An ally takes on the responsibility of learning about other identity groups’ experiences rather than putting the burden on individual members of these groups. To build empathy and anti‐racism, we offer resources in Table 1A for you to begin to learn more about the experiences of underrepresented groups. By constantly increasing your awareness of positionality, implicit biases, and microaggressions, you can reduce and prevent their impacts on underrepresented minority students in STEM.

## 4 NAVIGATING COMMON SOURCES OF URE ANXIETY

**FIGURE 2:**
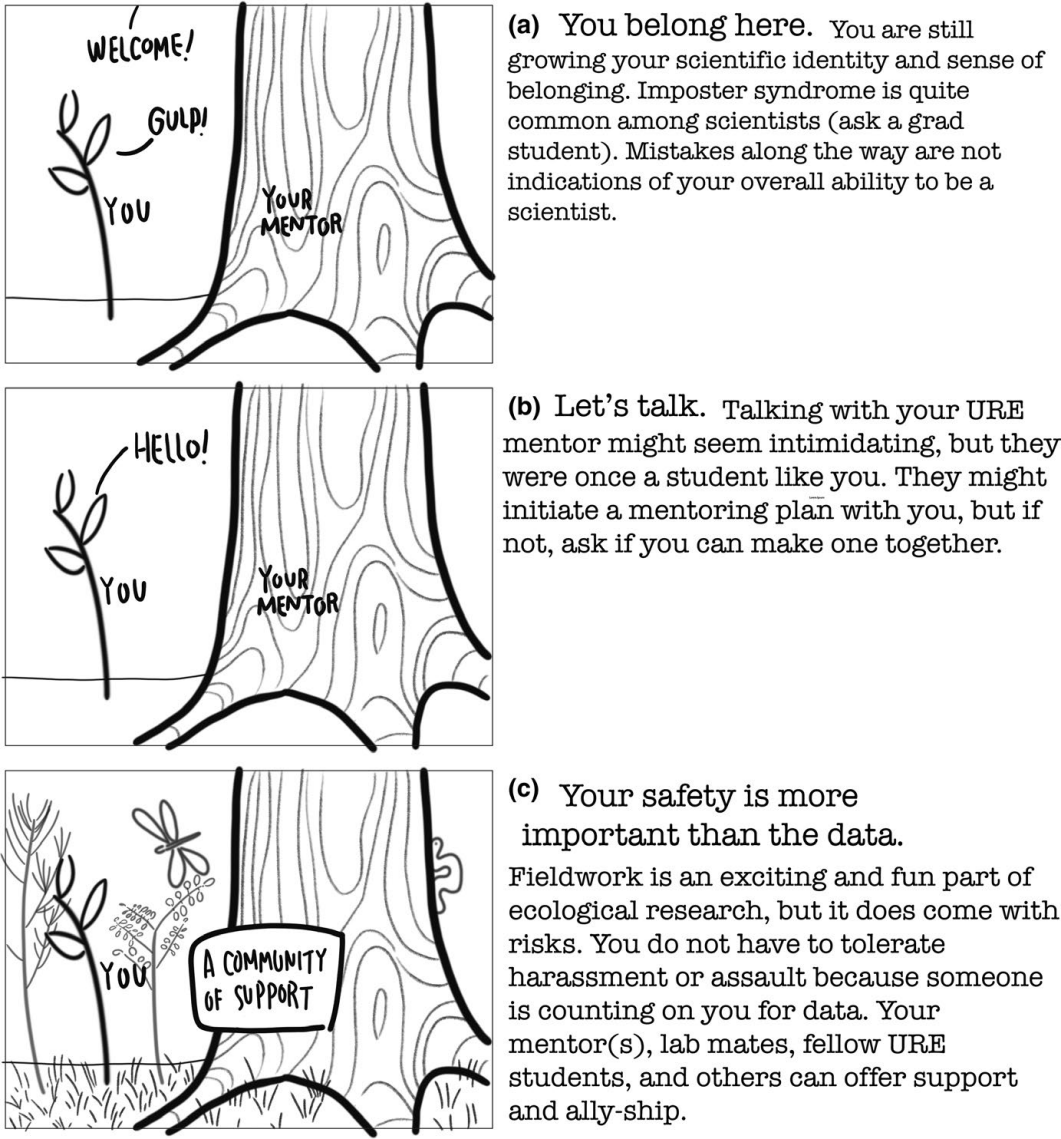
Strategies for undergraduate research experience (URE) students to successfully navigating three potential sources of anxiety. Original artwork

This section discusses three potential aspects of a URE where difficulty and/or anxiety can arise: imposter syndrome, communicating with your advisor, and fieldwork safety; we include strategies for navigating internally or externally stormy waters, should they occur. It is important to note that these three aspects are not unique to ecology and the strategies we provide can be translated to almost any professional setting. We proceed with another plant analogy: A URE student is like a seedling in the forest, where looking up at the size of your mentor trees can feel intimidating (Figure 2).

### 4.1 Imposter syndrome

As an undergraduate student, you are still building your scientific abilities, identity, and sense of belonging—things that take years of experience to build. Thus, lack of experience and confidence, feeling excluded (see above), and making mistakes can easily lead to feelings of inferiority or fraud (Figure 2a). If you have these feelings, *you are not alone*. This is a well‐known phenomenon termed “imposter syndrome.” Almost any scientist you meet, no matter their career stage, is dealing with or has dealt with this issue. Imposter syndrome is important to recognize because it can impact your mental health and is a known cause of students leaving STEM, especially students from underrepresented groups in STEM (Arnold et al., 2020; Cokley et al., 2017). There is no easy remedy for overcoming imposter syndrome, but a few things are known to help. First, knowing that others experience it can help put the feelings in perspective. While your feelings are real, they are not accurate portrayals of your ability to be a scientist. Second, its pervasiveness means imposter syndrome is ripe for discussion with your peers and lab group. Feel free to ask a graduate student and/or your project mentor, “Do you ever struggle with imposter syndrome?” Third, build a real‐life and virtual community of support (check Table 1B and 1C for some groups and organizations) (Halsey et al., 2020; Tseng et al., 2020). Also, know that failure is a necessary element to discovery and an excellent teacher. This is true in any STEM discipline and especially in ecology, where we learn from experiments involving many variables outside human control, such as weather. When failure happens, recognize it is not a reflection of you as a person or your scientific aptitude. Lastly, a powerful antidote to imposter syndrome is to celebrate all of your URE victories, no matter how seemingly small.

### 4.2 Communicating with your advisor

Your URE will be filled with new interpersonal relationships, including with your project mentor(s). A URE mentor can become a mentor for years to come, providing advice on next steps and writing letters of recommendation for you.

Navigating this new relationship can be unclear, especially if you are a first‐generation student. Communicating with your mentor can feel intimidating, whether they are a professor, postdoctoral researcher (person who has their PhD but is not yet a professor or other professional), or graduate student (Figure 2b). To ease the stress of communicating with your mentor, we recommend creating structure around these continuing conversations by developing a mentoring plan with your mentor (Emery et al., 2019; Masters & Kreeger, 2017; Montgomery, 2017). This plan can follow a formal format (e.g., an Individual Development Plan; https://myidp.sciencecareers.org/) or can simply be a bulleted list of important goals.

Realize that your mentor has many ongoing projects; communicating effectively with them will require you to initiate discussions on topics that are important to you (Questad & Knapp, 2007). As your URE progresses, be as open and honest as possible with your mentor about how your URE is going and what you hope to get out of it. After meetings, keep notes on your conversations to help you both remember what was discussed and agreed on.

### 4.3 Fieldwork

Ecological fieldwork is an incredible opportunity for personal growth, immersive and active learning, teamwork, and building friendships. Fieldwork is what draws many people to pursue careers in ecology. Students from underrepresented groups who complete a field‐based course, compared to those that do not, develop greater confidence, stay in their ecology and evolutionary biology major, complete college at higher rates, and finish college with higher GPAs (Beltran et al., 2020). Fieldwork associated with a URE could bring comparable results. However, you should know that the unique circumstances of fieldwork can result in distinct vulnerabilities. Although the vast majority of UREs will be both enlightening and safe, knowledge is power, and we want you to know harassment and assault can occur anywhere, including in the field (Archie & Laursen, 2013; Clancy et al., 2014; Nelson et al., 2017). Toxic field experiences can be devastating (Marín‐Spiotta et al., 2020; Nelson et al., 2017). Furthermore, individuals from underrepresented groups are more likely to experience harassment and assault in the field (Clancy et al., 2017).

What about fieldwork makes individuals especially vulnerable to harassment and assault? Fieldwork can mean unfamiliar cultural norms, long days of physically exhausting work, and harsh environmental conditions (ADVANCEGeo Partnership, 2019). Further, racism and heterosexism expressed by onlookers, property owners, or police heighten the risks of harassment and assault for Black and/or LGBTQ+ ecologists (Demery & Pipkin, 2020). It is the responsibility of the field team leader to cultivate a group dynamic that promotes and champions inclusive and equitable expectations for interpersonal behavior—including a code of conduct that addresses sexual harassment, discrimination, and bullying (Schneider et al., 2018) and a field risk management plan (Demery & Pipkin, 2020). As a URE student, you can ask for these resources and, in the absence of them, point to examples from ADVANCEGeo Partnership (2020) and considerations from Demery and Pipkin (2020).

#### 4.3.1 Know that your wellbeing and safety are more important than the data

If you experience harassment, assault, or anything unsafe, you do not have to tolerate it, period. Just because people are counting on you for data, does not mean you have to tolerate harmful experiences—forget the data. This sounds obvious, but in our experiences this is rarely made clear. If you experience harassment or assault, keep a written record of specific incidents, including the date, people present, and what was said or done. You have rights (e.g., Title IX and Title VI) and universities have special offices with names like “Office of Inclusion and Equity” or “Office of Institutional Opportunity and Access” for reporting discrimination, harassment, and assault. Keep in mind that the primary function of these offices is to make sure the institution complies with the law, and they are often not centered around the needs of the individual who experienced trauma (Cantalupo, 2019; Cantalupo & Kidder, 2019). If you are comfortable, speak to your URE mentor or another mentor or professor you trust. Postdoctoral researchers, graduate students, and your URE program director are also resources for support. Keep in mind, however, that professors and most university staff are mandatory (i.e., legally required) reporters of harassment and assault that occurs during university‐related activities or on university property. Counseling services are likely available at your URE or home institution in person or remotely—counselors are usually confidential, not mandatory reporters. Social media can also be a place to find support and solidarity (Table 1B). Consider finding an ally who can help you navigate the situation—you do not have to get through it alone (Figure 2c). Harassment and assault are *never* the fault of the person experiencing it.

In a perfect world, we would not need to write this subsection, but the ecology research culture is imperfect. As graduate students, postdocs, and career scientists work toward fixing it, we should not leave URE students like yourself in the dark. In fact, we hope that by building your awareness of the state of the culture, you develop an interest in joining the effort to improve the culture.

## 5 CONCLUSION

Here, we have explained, for the undergraduate reader, the scholastic and career benefits of pursuing a URE, noting that the URE is especially important for retaining diverse undergraduate researchers in ecology. We offered a primer of student practices that support inclusive laboratory culture according to different positionalities. With these practices and expectations, undergraduate researchers can help nudge the ecological research community toward greater diversity, equity, and inclusion. In the final section, we discussed three common sources of anxiety for undergraduate researchers and provided practical strategies for overcoming those anxieties. This paper is intended to break down barriers, so that all students can take on their URE with confidence, as an important and transformative step in their ecological pursuits.

We recognize that principal investigators and other URE mentors have greater power to change the structure of ecological research and culture. While a full overview of what can be done in that regard is beyond the scope of this paper, we provided suggestions and resources for initiating this work in Table 1 and Box 1. An inclusive and equitable ecology laboratory culture sets the stage for *any* URE student to transform from a student who knows science only through coursework to a confident field ecologist with a sense of belonging. This kind of change to the structure of ecological research and training is essential for building a community of ecologists that reflects the society we aim to serve.

## CONFLICT OF INTEREST

None declared.

## AUTHOR CONTRIBUTIONS

**Bonnie M. McGill:** Conceptualization (equal); investigation (lead); project administration (lead); resources (equal); supervision (lead); visualization (lead); writing – original draft (lead); writing – review and editing (lead). **Madison J. Foster:** Investigation (equal); project administration (supporting); resources (equal); writing – original draft (equal); writing – review and editing (equal). **Abagael N. Pruitt:** Investigation (equal); project administration (supporting); resources (equal); writing – original draft (equal); writing – review and editing (equal). **Samantha Gabrielle Thomas:** Investigation (equal); resources (equal); writing – original draft (equal); writing – review and editing (equal). **Emily R. Arsenault:** Investigation (equal); resources (equal); writing – original draft (equal); writing – review and editing (equal). **Janaye Hanschu:** Investigation (equal); resources (equal); writing – original draft (equal). **Kynser Wahwahsuck:** Investigation (equal); resources (equal); writing – original draft (equal). **Evan Cortez:** Investigation (equal); resources (equal); writing – original draft (equal); writing – review and editing (equal). **Kaci Zarek:** Investigation (equal); resources (equal); writing – original draft (equal); writing – review and editing (equal). **Terrance D. Loecke:** Conceptualization (equal); investigation (equal); resources (equal); writing – original draft (equal); writing – review and editing (equal). **Amy J. Burgin:** Conceptualization (equal); investigation (equal); resources (equal); writing – original draft (equal); writing – review and editing (equal).

## ETHICAL APPROVAL

No human or nonhuman animal subjects were used in this study.

## Data Availability

There are no data associated with this article.
